# An international survey of opinion regarding investigation of possible appendicitis and laparoscopic management of a macroscopically normal appendix

**DOI:** 10.1308/003588412X13373405385377

**Published:** 2012-10

**Authors:** SS Jaunoo, AL Hale, JPM Masters, SR Jaunoo

**Affiliations:** ^1^Warwickshire Surgical Research Group, University Hospitals Coventry & Warwickshire NHS Trust,UK; ^2^Heart of England NHS Foundation Trust,UK; ^3^University of Nottingham,UK

**Keywords:** Appendicitis, Laparoscopy, Diagnosis, Survey

## Abstract

**INTRODUCTION:**

The use of imaging and laparoscopy in patients with right iliac fossa pain as tools to make or exclude a diagnosis of appendicitis is at the discretion of the clinician. We sought to establish a consensus of opinion on this matter by surveying professional bodies for laparoscopic surgery in France, Italy and the US.

**METHODS:**

A survey was sent to members of the Society of American Gastrointestinal and Endoscopic Surgeons (SAGES), as well as the French Society for Endoscopic Surgery (SFCE) and the Italian Society for Endoscopic Surgery (SICE). The survey asked about management of both male and female patients presenting with right iliac fossa pain and what operative strategy the respondents would pursue should they find a macroscopically normal appendix at laparoscopy.

**RESULTS:**

A total of 364 responses were returned from the three groups. The responses from SAGES showed computed tomography to be the preferred modality for investigating patients with right iliac fossa pain, irrespective of sex. Both SFCE and SICE preferred the use of diagnostic laparoscopy, especially in the female patient group. The majority of all respondents stated that they would remove a macroscopically normal appendix at laparoscopy.

**CONCLUSIONS:**

Laparoscopy remains a potent tool in the management of appendicitis. However, the dilemma of when to remove a macroscopically normal appendix remains. Our study shows that removal of the appendix in this instance would be supported by an international consensus.

Despite its place as the archetypal surgical emergency, appendicitis often proves to be diagnostically challenging. This is particularly so in young women presenting with right iliac fossa pain. Increasingly, surgeons are turning to laparoscopy as both a diagnostic and therapeutic intervention for such patients.^1^ However, this technique may often raise questions of its own – specifically, what action to take when the appendix is deemed to be normal and no other pathology is found to account for the symptoms. The current literature fails to provide any clear guidance regarding this common scenario.

A 2009 study of members of the Association of Laparoscopic Surgeons of Great Britain and Ireland found that 61% would remove a ‘normal’ appendix and that 68% felt that guidance was needed.^2^ We sought to expand this survey of opinion by asking about the practices of members of the Society of American Gastrointestinal and Endoscopic Surgeons (SAGES), the French Society for Endoscopic Surgery (SFCE) and the Italian Society for Endoscopic Surgery (SICE).

## Methods

The administrators for SAGES, SFCE and SICE were contacted and kindly forwarded a survey (Table 1) to the members. There were no reminders and a response to the survey was not incentivised. Surveys sent to all groups were in English. Only fully completed surveys were included in the analysis. The results from questions 1–3 were analysed by simple descriptive statistics to illustrate the proportion of respondents that chose each management possibility. The answers to question 4 were analysed on a yes/no basis. Further details included in the responses to question 4 are not presented in the results.

**Table 1:** Questionnaire sent out to members of the Society of American Gastrointestinal and Endoscopic Surgeons, the French Society for Endoscopic Surgery and the Italian Society for Endoscopic Surgery

**Laparoscopy for right iliac fossa pain**
*Please highlight or bold the most appropriate answer*.
**1. In a young patient who presents acutely with right iliac fossa pain, with normal blood and urinalysis but who remains tender in the right iliac fossa, what would be your next management step?**
***Male***: Observation □ Ultrasonography □ Computed tomography □
Diagnostic laparoscopy □ Open appendicectomy □ Other □
***Female***: Observation □ Ultrasonography □ Computed tomography □
Diagnostic laparoscopy □ Open appendicectomy □ Other □
**2. You have made the decision to perform a diagnostic laparoscopy for your patient who has presented as a surgical emergency with right iliac fossa pain. Intra-operatively you find no pathology. Would you remove the appendix?**
***Male***: Yes No
***Female***: Yes No
**3. If yes, is this for any of the following reasons?**
To prevent future appendicitis For possible endoluminal appendicitis (inflammation of the mucosa of the appendix with an externally normal appendix) To avoid future confusion for the patient as to whether or not he or she has an appendix Other (please specify)
**4. Do you feel that there are sufficient clear guidelines on this topic?**
If so, from what source?

## Results

**Figure 1:**
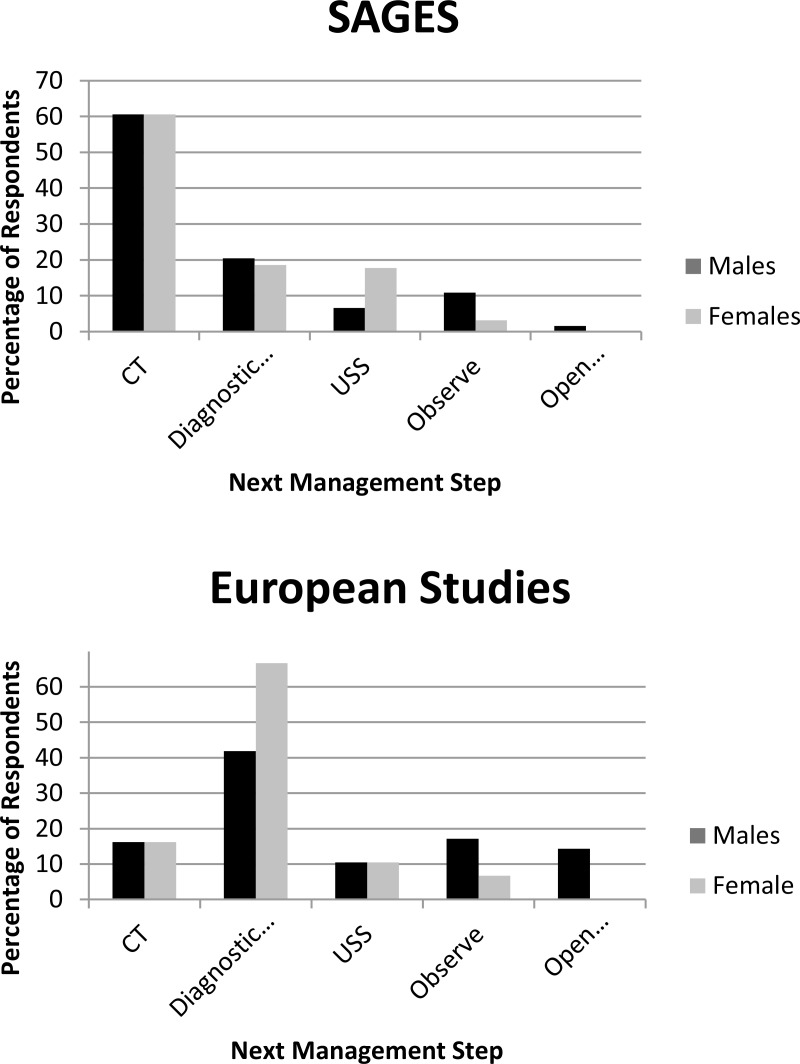
‘Next step’ management strategies for members of the Society of American Gastrointestinal and Endoscopic Surgeons (SAGES) (top) and the combined responses from the French Society for Endoscopic Surgery (SFCE) and the Italian Society for Endoscopic Surgery (SICE) (bottom)

A total of 259 responses were received from the members of SAGES, 79 from the members of SFCE and 26 from the members of SICE. The results are summarised in Figure 1.

Almost two-thirds (61%) of SAGES respondents would perform computed tomography (CT) for both male and female patients as their next management step while 15% would perform a diagnostic laparoscopy. Ultrasonography for both sexes was chosen by 7%, 5% would carry out a laparoscopy on male patients but ultrasonography on female patients, 4% would opt to observe male patients and request ultrasonography for female patients, 4% decided to observe male patients and carry out a laparoscopy on female patients, 3% would observe both sexes and 2% would perform an open appendicectomy on male patients while requesting ultrasonography for female patients.

Of the SFCE respondents, 44% would perform a diagnostic laparoscopy on both male and female patients as their next management step, 15% would choose CT for both sexes, 14% would observe male patients while opting for a diagnostic laparoscopy for female patients, 10% would request ultrasonography for both, 10% would perform an open appendicectomy on male patients while performing a diagnostic laparoscopy on female patients and 6% would observe both sexes.

A third of the SICE respondents (35%) would carry out a diagnostic laparoscopy on both sexes as their next management step, 27% would perform an open appendicectomy on male patients and a laparoscopy on female patients, 19% would request CT for both sexes, 12% would choose ultrasonography for both and 8% would observe both.

Of the respondents from SAGES, 69% would remove a normal appendix in male patients with 64% choosing to do so in female patients. For SFCE respondents, 68% would perform an appendicectomy in male patients and 71% in female patients while 73% of respondents from SICE would perform an appendicectomy in both sexes.

Respondents gave several reasons for performing an appendicectomy but by far the most common was ‘for possible endoluminal appendicitis’ (49%) followed by ‘to prevent future appendicitis’ (37%). The third most popular reason (15%) was ‘to avoid future confusion for the patient as to whether or not he or she has an appendix’.

Finally, 86% of respondents from SAGES felt that there was not clear guidance on the topic, as did 77% of SFCE respondents and 85% from SICE.

## Discussion

Appendicitis continues to pose a diagnostic challenge that is highlighted by the fact that 15–30% of appendices removed are macroscopically ‘normal’.^3^ The aim of this survey was to generate a body of opinion from expert groups regarding two key decisions that are made in the care of patients presenting with right iliac fossa pain.

The first decision step included in our survey was the investigation of a patient with right iliac fossa pain, normal blood tests and urinalysis results. There is no clear consensus of opinions, and stark differences existed between the European and American groups. American surgeons were overwhelmingly more likely to perform CT whereas the European societies favoured the diagnostic laparoscopy.

This is in contrast to the advice given by Lee *et al* who found that CT and ultrasonography had diagnostic accuracies of 74.5% and 43.4% respectively.^4^ They advised against the use of these imaging methods and warned that the time delay in waiting to obtain imaging may be detrimental. This division may reflect a difference in resource availability between the countries, for example if obtaining CT is not felt to delay the surgical intervention, or possibly a more aggressive approach from the European surgeons. It could also reflect the higher litigation rates in America compared with Europe, with American surgeons understandably wanting clear evidence for surgery before operating.

Culturally, it may be that the European surgeons tend to place more importance on factors such as cost and radiation dose as reasons for not opting for CT. An American study estimated that 29,000 future cancers could be related to CT performed in the US in 2007, with the biggest contributor being from abdominal and pelvic CT.^5^ Abdominal and pelvic CT accounts for 44% of the adult effective dose of radiation from all CT performed in the US.^6^ Cost is obviously highly pertinent in the minds of European surgeons given the current economic climate and proceeding to a diagnostic laparoscopy with its potential for intervention may be seen by these respondents as more cost effective.

A conservative management approach, as may be adopted in the UK in these circumstances, was very unpopular across all three countries surveyed. The explanation for this is uncertain but could be cultural, with British and Irish surgeons leaning more towards clinical assessment when making a decision and using imaging as a tool when the diagnosis is unclear.

The second strand to this survey was to seek consensus on whether an appendicectomy is appropriate at diagnostic laparoscopy in patients with right iliac fossa pain and a macroscopically normal appendix. This question was particularly pertinent to the European surgeons who opt to perform a diagnostic laparoscopy in 70% of female and 45% of male patients.

Between 64% and 73% of respondents cited that they would remove an appendix in this scenario. The advantages of performing an appendicectomy in this situation include early diagnosis of neoplasms, removal of endoluminal appendicitis, avoidance of confusion in patients who are unsure as to whether they have had their appendix removed and prevention of appendicitis in later life.^7–10^ To this end, many studies encourage the practice of removing a normal appendix. Garlipp and** Arlt argued that appendicectomy does not increase morbidity over a diagnostic laparoscopy and should therefore always be performed when acute appendicitis is suspected clinically.^7^ Chiarugi *et al* also support this argument, demonstrating a high false negative rate for diagnostic laparoscopy with pathological changes found in 58% of normal looking appendices removed.^11^

Nevertheless, much of the literature presents the alternative opinion. Champault *et al* recommended not removing macroscopically normal appendices due to the potential complications of an appendicectomy.^12^ They found morbidity occurred in 4.5% of patients undergoing a laparoscopic appendicectomy. Complications included abdominal abscess, post-operative ileus, wound infection, intra-operative bleeding and incisional hernias.^13,14^ Other studies have reported much higher rates, with Swank *et al* citing an intra-abdominal abscess rate of 6.2% after a laparoscopic appendicectomy.^13^ Nordenskjöld and Ahlgren found a history of appendicectomy to be more common in cases of ectopic pregnancy.^15^

Furthermore, there are the risks incurred from general anaesthesia and immobility following an operation. Van Dalen *et al* failed to find any evidence of increased long-term morbidity if an appendicectomy is not performed.^16^ Added to this are the economic considerations of removing a normal appendix and the additional operating time.

Our survey found that the majority of surgeons would remove a macroscopically normal appendix, which supports the practice of British and Irish surgeons where 61% would perform an appendicectomy.^2^ The most common reason given for removing the appendix was ‘for possible endoluminal appendicitis’. This has clear evidence behind it, with Phillips *et al* finding almost a third of apparently normal appendices being inflamed histologically.^17^ However, ‘to prevent future appendicitis’ also gained a large number of responses. This reason has little evidence in the literature, with the study by van Dalen *et al* showing no increase in long-term problems by leaving the appendix.^16^

The overwhelming majority of respondents (84%) felt there was a lack of clear guidance available on this common clinical scenario. Guidelines are used widely across the world and are useful to standardise practice as well as to ensure patients receive equal and optimal care. This lack of consensus therefore represents an issue for patient care.

## Conclusions

Based on the literature and the fact that almost a third of macroscopically normal appendices are found to be inflamed on histological analysis, we advocate that appendicectomy be performed in the absence of an alternative explanation for the patient’s symptoms. No conclusions can be drawn on the basis of this survey and the current literature regarding further investigation of the patient with right iliac fossa pain. It would seem that, for the near future, cultural and resource availability will have the largest bearing on the practices of surgeons in different parts of the world.
